# Unusual herpetic reactivation in a young female following botox injection: a case report study

**DOI:** 10.1186/s12879-023-08514-3

**Published:** 2023-10-02

**Authors:** Ehsan Amini-Salehi, Narges Eslami, Amirhossein Tamimi, Nasrin Sedighi, Saman Soltani Moghdam, Tofigh Yaghubi-Kalurazi, Soheil Hassanipour, Farahnaz Joukar, Fariborz Mansour-Ghanaei, Hojat Eftekhari

**Affiliations:** 1 Gastrointestinal and Liver Diseases Research Center, Guilan University of Medical Sciences, Rasht, Iran; 2 Guilan University of Medical Sciences, Rasht, Iran; 3 Department of Health, Nutrition & Infectious Diseases, School of Medicine, Guilan University of Medical Sciences, Rasht, Iran; 4 Department of Dermatology, School of Medicine, Guilan University of Medical Sciences, Rasht, Iran

**Keywords:** Varicella Zoster Virus, Herpes zoster, Shingles, Botulinum toxin, Botox, Case report

## Abstract

**Background:**

Botox injections are commonly used for cosmetic and therapeutic purposes because they temporarily paralyze muscles, reduce wrinkles, and alleviate certain medical conditions. Although generally considered safe and effective, Botox injections may cause potential complications. While herpes reactivation is more commonly associated with immunosuppressive therapies, such as chemotherapy or corticosteroid use, its association with Botox injection is poorly documented.

**Case Presentation:**

A 33-year-old woman presented with progressive painful rashes and vesicles on her forehead, scalp, and right upper eyelid, accompanied by fever and malaise following a Botox injection to treat wrinkles. A positive Tzanck smear test result confirmed the diagnosis of herpes infection. The patient was treated with antiviral medication, and her symptoms gradually regressed over several days.

**Conclusions:**

Although herpes reactivation is more commonly associated with immunosuppressive therapies, few cases of herpes zoster and herpes simplex following Botox injection have been reported. The pathogenesis of herpes reactivation following Botox injection is unclear; however, it has been hypothesized that the Botox protein is a potent antigen that may activate the cellular immune system, making it easier for the virus to reactivate. Healthcare providers should be aware of this potential complication and consider it when evaluating patients who present with painful rashes following Botox injections. In addition, individuals who want to receive Botox injections should be informed of this complication. The diagnosis of herpetic infection should be made promptly, and antiviral therapy should be initiated to minimize the risk of complications. Further research is needed to better understand the pathogenesis and risk factors for herpes following Botox injection and to develop strategies for preventing and managing this complication.

## Introduction

Varicella-zoster virus (VZV) is a member of the Herpesviridae family, including herpes simplex virus types 1 and 2, Epstein-Barr virus, and cytomegalovirus [1]. The virus is highly contagious and can be transmitted by direct contact with fluid from the rashes of an infected person or by airborne droplets [2–4]. VZV causes two distinct clinical syndromes: chickenpox (varicella) and shingles (herpes zoster). Chickenpox is the primary infection manifestation in children with itchy blisters and disseminated rashes [5]. The virus then can become latent in the peripheral nervous system and reactivated, resulting in shingles in adulthood [6].

Botulinum toxin (Botox) injection is a popular cosmetic treatment for wrinkles but is also used for various medical conditions such as migraines, cervical dystonia, and hyperhidrosis [7–10]. Based on immunological properties, molecular size, biosynthesis, and cell mechanisms, botulinum toxin is divided into eight serotypes (A, B, C1, C2, D, E, F, and G)[11]. Only type A and B of these have been commercialized for use in clinical settings [12]. Botox works by blocking the release of acetylcholine, a neurotransmitter that causes muscle contractions, leading to muscle relaxation and smoother skin. However, Botox injection can potentially cause adverse effects [13].

Recent studies have suggested a possible association between herpetic infection and Botox injection. VZV reactivation has been reported in patients following Botox injections for cosmetic and medical purposes [14, 15]. The mechanisms behind this association are not entirely clear, but it is thought that Botox may disrupt the normal immune response, leading to VZV reactivation in susceptible individuals [16–19].

In the present study, we introduce a young woman who developed shingles following Botox injection, and by expressing an opinion, we attempt to support the idea that cellular immunity is the underlying cause of both events.

## Case presentation

A 33-year-old woman presented to our hospital with vesiculopustular lesions following a cosmetic Botox injection on March 23, 2023. She stated that Botox was injected into forehead lines, crow’s feet lines, and the vertical ‘11’ frown lines between the eyebrows five days before her referral to our hospital.

The patient’s medical history seems relatively unremarkable, exception for having had chickenpox at the age of 8, without any history of sore cold in anywhere of body. There were no records of any medication usage, allergic reactions, or past surgeries. Moreover, there were no known hereditary diseases within her family.

**Fig. 1:**
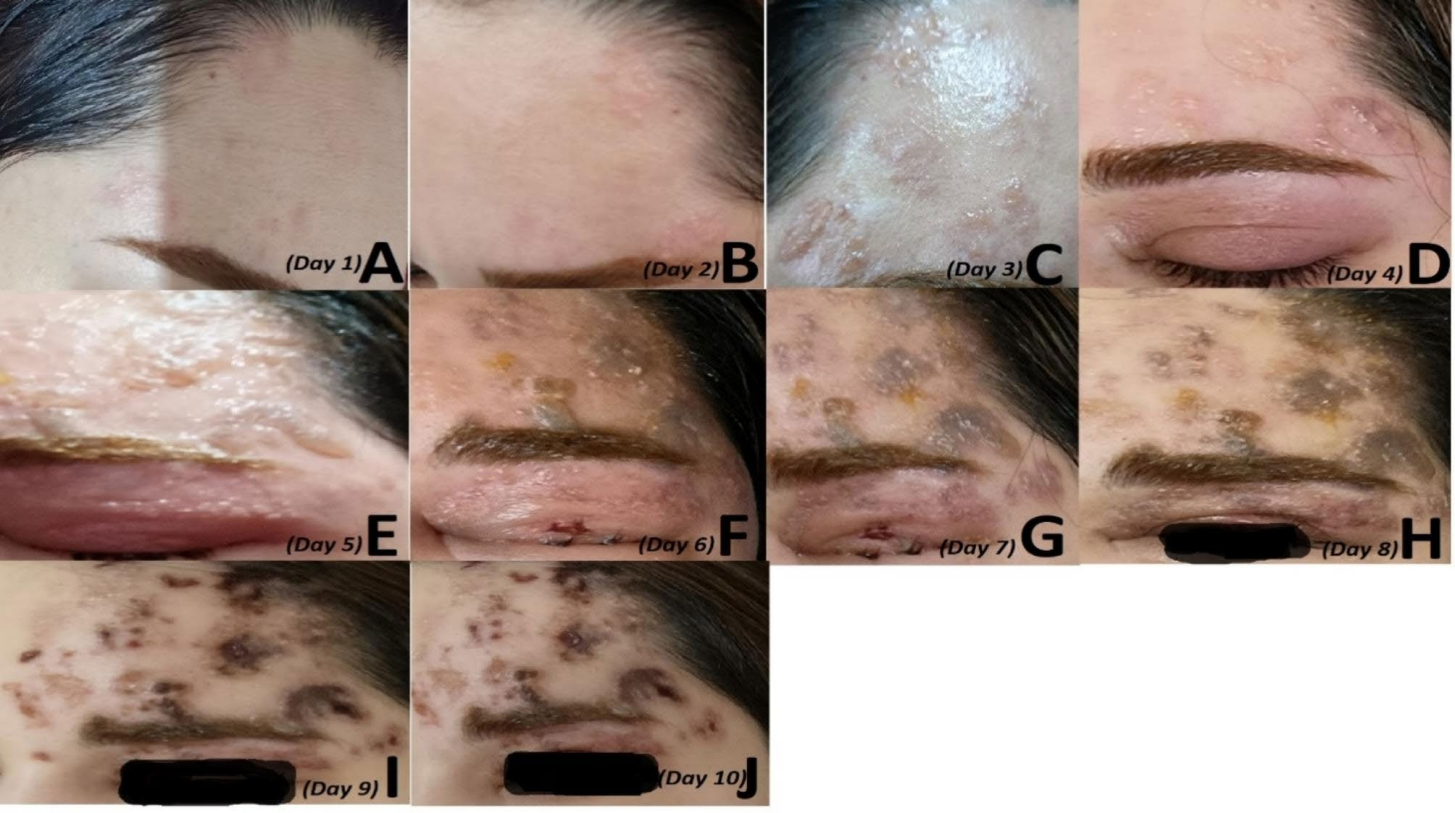
Patients lesions after Botox injection (A-E) and after intravenous acyclovir initiation *(F-J*)

The patient reported that within the first 24 h after injection, she felt considerable pain and a burning sensation in the injected areas. The next day, the first lesions appeared as inflammatory vesicles in the right frontal region (Fig. 1A). On the second day, the patient reported feeling fever and chills, and the lesions developed (Fig. 1B). On the third day, vesiculopustular lesions appeared (Fig. 1C). On the fourth day, preorbital inflammation occurred along with vesiculopustular lesions progression (Fig. 1D). On the fifth day (the day she referred us), the patient had various vesiculopustular lesions and preorbital edema (Fig. 1E).

**Fig. 2:**
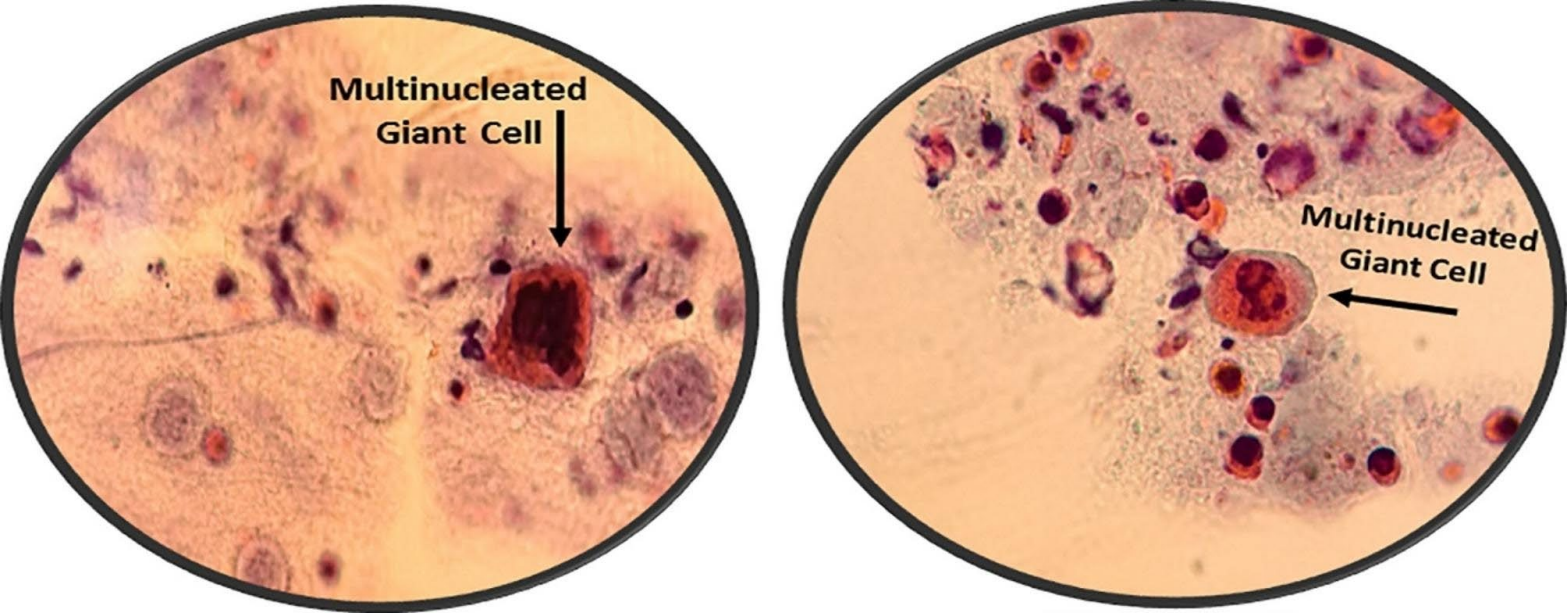
The Tzanck smear test of the patient showing multinucleated giant cells which are a hallmark for herpetic infection

Initial evaluations were done for her, and since we were suspected of herpetic infection, a Tzanck smear test was conducted for her, which was suggestive of herpetic infection (Fig. 2). We also conducted a Human Immunodeficiency Virus antibody (HIV Ab) test to rule out immunodeficiency, and the result returned negative. We started intravenous acyclovir and followed the patient. In the following days, the vesiculopustular lesions regressed and became crusted (Fig. 1F-J). In addition, the preorbital edema improved, as illustrated in figures F-J. The patient’s signs and symptoms, including fever, chills, and pain, disappeared gradually after initiation of treatment.

## Discussion

Following the initial infection by the herpes virus, the virus stays dormant in the peripheral autonomic ganglia [20]. Herpes zoster results from virus reactivation which is presented as pain, pruritis, dysesthesia, and malaise, followed by unilateral dermatomal vesicles, which get ulcerated and crusted [21]. Herpes zoster can also be manifested as vision change, headache, earache, and dysgeusia when it involves the face. It rarely leads to encephalitis, pneumonia, and bacterial skin superinfection [21]. Moreover, it can be accompanied by different neurological syndromes, including postherpetic neuralgia (PHN), the major complication of herpes zoster [21].

The presenting study reported a herpetic infection following the first cosmetic Botulinum Toxin Type A (BTX-A) injection in a young, healthy woman who responded well to acyclovir. Similar to our case, Graber et al. [14] reported two cases of herpes zoster infection after cosmetic Botox injections. Their patients were older than ours and showed lesions almost one week after injection, while in our case, vesicles developed one day after injection. In another study by de Souza et al. [15], a 43-year-old woman developed herpes zoster after Botox injection. The patient first developed inflammation and erythematous papules, which misled the physicians to diagnose the condition as cellulitis; however, after vesicle development and no response to antibiotic therapy, the herpes zoster diagnosis was made. The reactivation of VZV after Botox injection for medical treatment is also reported in conditions like migraine and hyperhidrosis (Table 1) [22–24].

**Table 1:** Previous reports of Herpes zoster development following BTX-A injection

Author	Patients Age (Year)	Injection location	Injection Indication	Patient’s risk-factors	Complication
de Souza et al. [15]	43	Forehead, glabella, and lateral periorbital areas	Cosmetic	None	None
Farahmand et al. [22]	68	Forehead, glabella, and periorbital areas	Blepharospasm	Old age Hypertension	Encephalitis
Gadient et al. [23]	72	Periorbital areas	Migraine	Old age Previous Herpes zoster	Minor dysesthesia Following resolution
Graber et al. [14]	55	Glabella, forehead, and lateral periorbital areas	Cosmetic	None	None
	48	Glabella, forehead, and lateral periorbital areas	Cosmetic	None	Persistent subacute headache
Muzumdar et al. [24]	25	Axilla	Hyperhidrosis	None	None
Ramappa et al. [39]	55	periocular	eyelid disorder, management of spastic entropion	Previous viral keratitis	Corneal abrasions with a spastic entropion
Narang et al. [40]	59	lacrimal gland	epiphora due to intractable lacrimal disorders	Previous herpes simplex viral keratitis	Watering and discomfort in both the eyes and severe ptosis

BTX-A is the most potent member of botulinum neurotoxins [25]. It was approved by the Food and Drug Administration (FDA) for different therapeutic and cosmetic purposes, including treating blepharospasm, hemifacial spasm, muscle relaxation, and glabellar wrinkles [13, 25]. It also reduces specific nociceptor-sensitizing chemicals to reduce neuroinflammation [25].

Botulinum neurotoxins are composed of a core neurotoxin and complexing accessory clostridial proteins; however, due to the purifying process, these proteins’ complexity has been reduced [18]. Such complex proteins have been considered possible immune cell stimulators. So, as antigens, the proteins in botulinum toxin can trigger an immunological response promoting the production of antibodies and cytokines, which sometimes leads to failure of Botox treatment [18, 26, 27].

According to previous reports, complications arising from Botox injections have been associated with the reactivation of herpes simplex virus (HSV) and varicella-zoster virus (VZV). A comprehensive review of the patient’s medical history, including the location of virus reactivation, the types and stages of vesicular lesions, the severity of the recurrent disease, and the results of the Tzanck test, led to the consideration of herpes zoster as a possible diagnosis, given the absence of periodic subclinical reactivation.

The pathophysiology of herpetic reactivation has yet to be fully understood [14]. However, it is supposed to be related to reducing VZV-specific cellular immunity [28]. Immunodeficiency, systemic diseases, and cancers, along with old age, are shown to be risk factors for Herpes zoster development [15]. Besides, various factors can trigger VZV reactivation, including radiotherapy, skin graft, ophthalmic laser procedures, dental procedures, and surgeries [23].

**Fig. 3:**
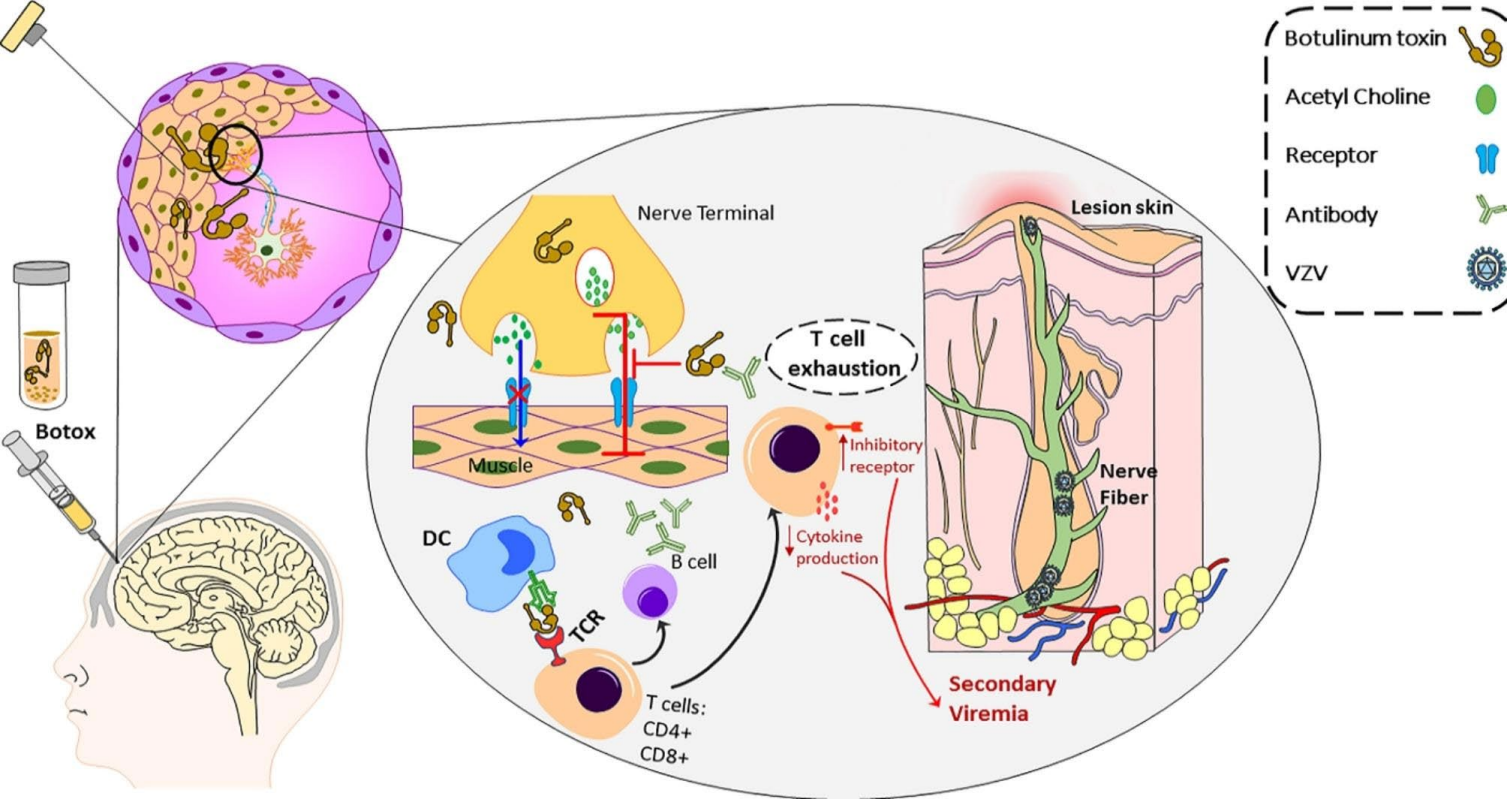
Schematic picture of the immune process during Botox’s simultaneous injection and the varicella-zoster virus’s reactivation. The amount of protein that dendritic cells pick up from a formulation of botulinum toxin and present to T cells via their particular receptor (TCR) determines antigenicity. Conversely, once T cells experience prolonged antigen exposure, CD4 + and CD8 + T cell production declines, and T cell exhaustion occurs due to certain epigenetic variables. VZV initiates to reactivate following CD4 + and CD8 + T reduction. (VZV, varicella zoster virus; DC, Dendritic cells; TCR, T-cell receptor)

It seems that the relation between Botox injection and herpes reactivation can be attributed to the alteration of the cellular immune system through T cells. T cells that experience prolonged or high doses of antigen exposure (including Botox complex proteins) lose their capacity to function efficiently. This phenomenon, known as T cell exhaustion, is attributed to atypical immunological checkpoint protein expression (Fig. 3) [29–31].

One essential element in the T cell exhaustion process is a transcription factor known as thymocyte selection-associated HMG BOX (TOX). Sustained TOX expression changes some epigenetic factors, inhibits cell differentiation, and enters a state of T cell exhaustion [32]. TOX expression causes downregulation of CD4 + and CD8 + T cells [33]. By over-expression of TOX and the following T cell exhaustion, the production of Tumor Necrosis Factor α (TNF-α), Interleukin 6 (IL-6), and Interferon *α* (IFN- α) decreases [32, 34, 35], resulting in reactivation of the virus [36, 37]. In contrast, in latently infected persons, T-cell responses were induced by the expression of T-cell cytokines such as Tumor necrosis factor α (TNF-α), interleukin 6 (IL-6), and Interferon *α* (IFN- α) within both CD4 and CD8 subsets.

[28, 36]. These cytokines correlate with mild varicella, whereas cell-mediated immunodeficiency (low and depleted VZV-specific CD4 and CD8 T-cell responses) may also be associated with severe shingles (Fig. 3) [38].

### Limitations and future suggestions

In the current study, we presented a rare case report of herpetic reactivation following Botox injection; however, we acknowledge several limitations that need to be considered. Firstly, the study is based on a single case report of a 33-year-old woman who developed herpes zoster following Botox injection. Consequently, caution should be exercised when attempting to generalize these findings to a broader population, as individual variations may exist. Furthermore, establishing a definitive causal relationship between Botox injections and the development of shingles may prove challenging due to the limited reports in this regard. Further cross-sectional and meta-analysis studies are needed to evaluate this relationship.

Another limitation is the absence of a long-term follow-up for the patient. After the resolution of symptoms and improved dermal lesions, the patient was discharged, leaving us uncertain about any potential herpetic reactivation risk during her lifetime. Recognizing this knowledge gap and further investigating the potential long-term implications of VZV reactivation following Botox injections is essential.

Moreover, while orbital edema showed gradual improvement following the initiation of treatment, we inadvertently neglected to have an ophthalmologist assess the patients. It is crucial to ensure proper evaluation by a specialist in future cases.

We lacked specific information regarding the brand and quality of the injected Botox. Variations in Botox formulations or administration techniques could impact the outcomes, making it crucial for future studies to address this aspect for a more comprehensive understanding.

In addition, it is essential to acknowledge that genetic factors can significantly influence the immune system’s response to viral reactivations. The interplay between genetic predispositions and immune system functionality varies among individuals, leading to diverse outcomes when faced with infections like herpes zoster.

## Conclusion

Botox injection as a cosmetic and treatment procedure is famous among individuals hence knowing the possible complications is essential for medical staff and patients. Herpetic reactivation is one of the side effects which can be misdiagnosed with other conditions like bacterial infections. Medical staff who offer cosmetic services should consider this condition and inform their patients about the potential risks associated with herpetic reactivation. Educating patients about the possibility of developing skin lesions following herpetic reactivation is crucial. By providing this essential information, patients can be vigilant and promptly seek medical attention if they notice any suspicious lesions or skin changes. Early recognition of the herpetic infection with prompt initiation of antiviral therapy is crucial in preventing the progression of skin lesions and promoting the patient’s recovery. Furthermore, it is essential to conduct HIV-related tests to evaluate potentially acquired immunodeficiencies in the presence of dermatological herpes.

## Data Availability

The data of the current study are available on reasonable request from the corresponding author.

## Abbreviations

VZV: Varicella-zoster virus
PHN: postherpetic neuralgia
BTX-A: Botulinum Toxin Type A
FDA: food and drug administration
TOX: thymocyte selection-associated HMG BOX
TNF-α: Tumor necrosis factorα
IL-6: Interleukin 6
IFN-α: Interferonα
DC: Dendritic cells
TCR: T-cell receptor
